# Wie wird die EU-Verordnung zu klinischen Arzneimittelprüfungen ein Jahr nach ihrem Start bewertet?

**DOI:** 10.1007/s00103-022-03634-8

**Published:** 2022-12-13

**Authors:** Thomas Sudhop, Hartmut Krafft

**Affiliations:** 1 Bundesinstitut für Arzneimittel und Medizinprodukte (BfArM), Kurt-Georg-Kiesinger-Allee 3, 53175 Bonn, Deutschland; 2 Paul-Ehrlich-Institut (PEI), Bundesinstitut für Impfstoffe und biomedizinische Arzneimittel, Paul-Ehrlich-Str. 51–59, 63225 Langen, Deutschland

Zum **31.01.2022** wurde die Verordnung (EU) Nr. 536/2014 über klinische Prüfungen (EU-Verordnung) anwendbar. Ab diesem Datum konnten Antragstellerinnen und Antragsteller erstmalig eine Genehmigung für die Durchführung von klinischen Arzneimittelprüfungen gemäß der EU-Verordnung beantragen und Erfahrungen mit der EU-Verordnung und den zugehörigen IT-Systemen sammeln.

Die EU-Verordnung hat die Intention, das Autorisierungsverfahren klinischer Prüfungen europaweit zu harmonisieren und zu beschleunigen. So wurde das Antragsverfahren einschließlich der einzureichenden Unterlagen standardisiert und eine vollständig elektronische Unterlageneinreichung und Kommunikation zwischen allen Verfahrensbeteiligten über ein Internet-basiertes EU-Portal eingeführt. Das *Clinical Trials Information System *(CTIS), das von der Europäischen Arzneimittel-Agentur (EMA) entwickelte und betriebene IT-System, ist für das Funktionieren der Prozesse und den Informationsaustausch unter den Beteiligten essentiell.

Während des ersten Jahres nach Anwendbarkeit der EU-Verordnung können Sponsoren entweder das neue harmonisierte Antragsverfahren gemäß EU-Verordnung nutzen oder noch Anträge nach den bisherigen nationalen Verfahren auf Basis der europäischen Richtlinie 2001/20/EG stellen. Mit dem **31.01.2023** endet die letztgenannte Möglichkeit. Ab diesem Datum können Neuanträge nur noch gemäß der EU-Verordnung gestellt werden. Bereits laufende klinische Prüfungen, die noch gemäß der Richtlinie und den nationalen Regularien autorisiert wurden, können noch bis zum 30.01.2025 im alten nationalen rechtlichen Kontext fortgeführt werden.

Bereits im Jahr 2017 beschäftigte sich in Heft 60 eine gesamte Ausgabe des *Bundesgesundheitsblatts* mit der europäischen Neuregelung klinischer Arzneimittelprüfungen. Im vorliegenden Heft berichten nun die verschiedenen Stakeholder klinischer Prüfungen über ihre ersten Erfahrungen mit der EU-Verordnung.

Im ersten Beitrag beschreiben *Sudhop et al.* die Grundzüge des neuen Genehmigungsverfahrens und der Zusammenarbeit zwischen den Mitgliedstaaten. So erlaubt die koordinierte gleichzeitige Bewertung von multinationalen Anträgen durch alle beteiligten Mitgliedstaaten eine schnellere und einfachere Genehmigung besonders dieser klinischen Prüfungen. Trotz der weitgehenden Harmonisierung des Verfahrens erlaubt die EU-Verordnung noch vereinzelt nationale Besonderheiten bzw. lässt einige operative Elemente ungeregelt, wie z. B. die Beteiligung nationaler Ethikkommissionen, so dass in allen Mitgliedstaaten neben der EU-Verordnung noch nationale Umsetzungsregelungen erforderlich sind, die *Frech und Kolleginnen und Kollegen* in ihrem Beitrag für Deutschland erläutern.

*Chase und Kolleginnen* beschreiben in ihrem Beitrag erste Erfahrungen mit der EU-Verordnung aus der Sicht von Auftragsforschungsinstituten, welche Sponsoren unter anderem auch bei der Antragstellung klinischer Prüfungen unterstützen und daher auch bereits vermehrte Erfahrungen mit CTIS und den Prozessen sammeln konnten. In ihrem Artikel adressieren die Autorinnen u. a. die Komplexität des Rollen- und Rechtesystems von CTIS und beschreiben mögliche Vorgehensmodelle in der Zusammenarbeit zwischen Sponsor und Auftragsforschungsinstitut bei der Antragstellung über CTIS. *Henn und Ruppert* analysieren die Chancen, aber auch die Herausforderungen der EU-Verordnung aus Sicht der forschenden pharmazeutischen Industrie. In ihrer Analyse resümieren sie den Stand der Vorbereitung in Deutschland, einschließlich des nationalen Pilotprojekts. Als immer noch problematisch werden von ihnen die nationalen Regelungen zum Strahlenschutz angesehen, die weiterhin ein nationales Anzeige- bzw. Genehmigungsverfahren beim Bundesamt für Strahlenschutz erforderlich machen, welches mit dem Genehmigungsverfahren der EU-Verordnung synchronisiert werden muss.

Während einige der Prozesse der EU-Verordnung für die pharmazeutische Industrie teilweise vertraut sind, stellt die Arbeit mit CTIS und seinen Um-Systemen akademische Forschungsteams vor neue Herausforderung, die *Fuhrmann und Kolleginnen* in ihrem Beitrag beschreiben. Neben Erschwernissen durch z. B. neue Datenbanken beschreiben die Autorinnen weitere, für akademische Studien neue Anforderungen, die die Planung und Durchführung akademischer Studien aus ihrer Sicht kaum – wie eigentlich von der EU-Verordnung intendiert – erleichtern.

Auch für die Forschungsethikkommissionen, die nun auf der Basis der EU-Verordnung Teile der Unterlagen klinischer Prüfungen gemeinsam mit der jeweils zuständigen Bundesoberbehörde bewerten, aber weiterhin wie bisher die Eignung von Prüfern und Prüfzentren sowie die an die Studienteilnehmerinnen und -teilnehmer gerichteten Unterlagen einschließlich Unterlagen zu Datenschutz und Probandenversicherung in eigener Zuständigkeit bewerten, sind die prozessualen und administrativen Anforderungen deutlich gestiegen, wie *Raffauf-Seufert und Grass* in ihrem Beitrag beschreiben.

Die EU-Verordnung schafft eine deutlich höhere Transparenz klinischer Prüfungen. So werden mit wenigen Ausnahmen alle über CTIS übermittelten Dokumente – zeitlich gestaffelt und teilweise geschwärzt – öffentlich einsehbar. Dabei kommt dem Schutz von Betriebs- und Geschäftsgeheimnissen eine besondere Bedeutung zu, die die verschiedenen Interessengruppen unterschiedlich bewerten. So formuliert *Strech* in seinem Beitrag aus Sicht unabhängiger Forschungsgruppen zum einen Lob bzgl. der stärkeren Transparenz an sich, zum anderen aber auch Kritik an einer teilweise zu späten und nicht weitgehenden Veröffentlichungspolitik der EMA, die unabhängige Forschungsteams in ihrer Arbeit einschränke. Umgekehrt bewerten *Wachenhausen und Peters* die Transparenzregelungen aus Sicht der pharmazeutischen Unternehmen teilweise kritisch und formulieren sogar ihre Sorge um mögliche Abwanderungstendenzen klinischer Forschung aus der EU. Im letzten Heftbeitrag analysieren *Klingmann und Kolleginnen und Kollegen* schließlich die Neuerungen der EU-Verordnung aus Sicht von Patientinnen und Patienten und deren Organisationen. Stellvertretend wünschen sich die Autorinnen und Autoren eine weitergehende Beteiligung von Patienteninteressengruppen, z. B. auch bei der Bewertung in Ethikkommissionen, sowie eine schnellere Verfügbarkeit der für Patientinnen und Patienten relevanten Informationen.

Als Editoren dieses zweiten Themenheftes zur EU-Verordnung über klinische Arzneimittelprüfungen freuen wir uns, den Leserinnen und Lesern mehr als 5 Jahre nach dem ersten Heft nunmehr den aktuellen Sachstand nach Beginn der Anwendbarkeit der EU-Verordnung aus der Perspektive der verschiedenen Beteiligten zu präsentieren.

Wir wünschen Ihnen eine interessante und anregende Lektüre.

